# Real-time Characterization of Antibody Binding to Receptors on Living Immune Cells

**DOI:** 10.3389/fimmu.2017.00455

**Published:** 2017-04-24

**Authors:** Sina Bondza, Eleanor Foy, Jonathan Brooks, Karl Andersson, James Robinson, Pascale Richalet, Jos Buijs

**Affiliations:** ^1^Ridgeview Instruments AB, Vänge, Sweden; ^2^Department of Immunology, Genetics and Pathology, Uppsala University, Uppsala, Sweden; ^3^Leeds Institute of Rheumatic and Musculoskeletal Medicine, University of Leeds, Leeds, UK; ^4^Pfizer Inc., Cambridge, MA, USA; ^5^BioRevera LLC, Arlington, MA, USA

**Keywords:** affinity, kinetics, therapeutic antibody, B-cells, T-cells, CD20, Fcγ receptor

## Abstract

Understanding molecular interactions on immune cells is crucial for drug development to treat cancer and autoimmune diseases. When characterizing molecular interactions, the use of a relevant living model system is important, as processes such as receptor oligomerization and clustering can influence binding patterns. We developed a protocol to enable time-resolved analysis of ligand binding to receptors on living suspension cells. Different suspension cell lines and weakly adhering cells were tethered to Petri dishes with the help of a biomolecular anchor molecule, and antibody binding was analyzed using LigandTracer. The protocol and assay described in this report were used to characterize interactions involving eight cell lines. Experiments were successfully conducted in three different laboratories, demonstrating the robustness of the protocol. For various antibodies, affinities and kinetic rate constants were obtained for binding to CD20 on both Daudi and Ramos B-cells, the T-cell co-receptor CD3 on Jurkat cells, and the Fcγ receptor CD32 on transfected HEK293 cells, respectively. Analyzing the binding of Rituximab to B-cells resulted in an affinity of 0.7–0.9 nM, which is similar to values reported previously for living B-cells. However, we observed a heterogeneous behavior for Rituximab interacting with B-cells, which to our knowledge has not been described previously. The understanding of complex interactions will be facilitated with the possibility to characterize binding processes in real-time on living immune cells. This provides the chance to broaden the understanding of how binding kinetics relate to biological function.

## Introduction

The human immune system is a complex network of cells, which communicate with each other in a highly organized manner to defend the body from pathogens and other potentially harmful substances. One way in which immune cells communicate is through secreted molecules that bind to designated receptors on target cells. The recognition of a pathogen or another ligand by a cell-surface receptor leads to an intracellular signaling cascade, which alters the immune cell’s behavior in response to the received stimulus. The effect triggered by a molecular interaction is not only dependent on the molecules engaged but is also affected by the affinity, avidity, and kinetics of the interaction.

A well-known example of this is the positive and negative selection of T-cells in the thymus; the fate of the thymocyte is dependent on how well the T-cell receptor binds self-antigens presented *via* the MHC of antigen presenting cells. A high affinity interaction with self-antigens will lead to apoptosis, whereas a weak affinity will induce survival signals and promote positive selection (1). In this case, interactions of structurally very similar molecules can lead to completely opposing outcomes depending on the strength of the interaction. Therefore, a detailed characterization and quantification of a molecular interaction is required for an in-depth understanding of immune cells interacting patterns.

Apart from broadening our knowledge of physiological interactions, affinity and kinetics are also crucial when it comes to drug development (2). The fastest growing class of pharmaceuticals is the one of monoclonal antibodies (mAbs) (3). The first approved mAb in 1986 was Muromonab, used for the treatment of renal graft rejection. Muromonab acts as an immunosuppressor and binds to CD3, thereby inhibiting signaling and activation of T-cells (4). Since then, most of the developed mAbs have been for applications in oncology and autoimmunity (4). Their effects are partially mediated by the variable region binding to an epitope expressed on cancer cells and thus modifying the signaling mediated *via* the receptor, usually resulting in growth arrest or apoptosis (5). However, it has become increasingly apparent over the last few decades that the clinical effectiveness of mAbs is also due to interaction with the immune system *via* the Fc part of the mAb. In a process termed antibody-dependent cell-mediated cytotoxicity, the Fc part of cell-bound mAbs is recognized by Fcγ receptors on NK cells, which ultimately leads to lysis of the tumor cell (6). In addition, complement-dependent cytotoxicity (CDC) is a suggested mechanism of action for mAbs (7) as shown for Rituximab (8).

Rituximab was approved by the FDA in 1997 as the first mAb for cancer therapy. It works by binding to the B-cell marker CD20 causing depletion of both malignant and normal B-cells (9). Due to its success in treating various B-cell malignancies (10, 11), second-generation anti-CD20 mAbs have been developed with improved properties (12, 13). For example, Ofatumumab, which is also an anti-CD20 mAb, exhibits an increased ability to induce CDC compared to Rituximab (14). It is thought that the redistribution of mAb-bound CD20 into lipid rafts plays a role in inducing CDC, and in an *in vitro* study, stronger CDC effects were correlated with slower off-rates of the tested mAbs (14). However, in a follow-up study these observations were challenged (15), and the role in which anti-CD20 off-rate contributes to lipid raft formation and CDC is debated (16, 17).

The effort to try and understand how kinetics relate to biological function is important, since this knowledge would help tailoring the design and selection of next generation mAbs (18). Due to the biological complexity of many interactions that are influenced by contributing co-receptors, receptor oligomerization, and clustering, it is advantageous to measure interactions on the intended target cell type (19, 20).

There are many techniques available to study interactions between drugs and their targets (21) of which a number are suitable to not only study the affinity but also the kinetics. Some biophysical techniques, such as surface plasmon resonance (SPR) (22), biolayer interferometry (BLI) (23), and the quartz crystal microbalance (QCM) (24), have been applied on interaction measurements where the target is in or on a cell. The measurement principle is either based on ligand binding induced changes in the refractive index in close proximity to a surface (SPR and BLI) or changes in the vibration frequency (QCM). A number of studies using living cells have been performed generating interesting correlations between ligand binding and overall cellular responses in a dose- and compound-dependent manner (25–27). To extract the interaction rate constants and the affinity from a real-time interaction measurement, however, the signal needs to be proportional to the number of bound complexes. A commonly used approach to minimize signals originating from density fluctuations of cells is to fixate them (28–31). With fixated cells, however, one risks missing the real-life complexity of a living cell such as ligand binding induced clustering and dimerization of receptors (20, 32). Other aspects, such as sterical hindrance in antibody binding to the epidermal growth factor receptor 2 by mucins could be assessed (28). For interaction measurements on living cells, fluorescence-activated cell sorting (FACS) is commonly used, particularly in the area of immunology. Although FACS is mostly used for end-point assays, a number of studies reported dynamic interaction analysis by sampling from a larger reaction vessel at various time points (33, 34). As FACS is primarily designed for end-point measurements for cells in suspension, it lacks the possibility for rapid changing from ligand incubation to monitoring dissociation as removal of the ligand typically involves a centrifugation and suspension step. It also lacks simultaneous monitoring of non-specific interactions of the ligand to a reference area as commonly applied in instrumentation designed for real-time interaction analysis (35).

A method designed to measure ligand–receptor interactions in real-time on living cells is LigandTracer (36). From the resulting binding trace, kinetic parameters such as on- and off-rates and the affinity can be extracted. The shape of the binding trace also indicates if the interaction follows a simple “one-to-one” pattern, in which one ligand binds to one receptor in one defined way, or if the interaction is more complex. A prerequisite for the assay is that the cells stay attached in a confined area on the cell dish.

As reported by the literature, LigandTracer measurements have almost exclusively been conducted on adherent growing cells. A protocol based on EDC/NHS [1-ethyl-3-(3-dimethylaminopropyl)carbodiimide hydrochloride/*N*-hydroxysuccinimide] coupling for attachment of suspension cells has been applied (37, 38) but proved effective only in a few cases. In this study, we have developed a method that allows suspension cells to be tethered in a confined area on a cell dish for a feasible measurement time of several hours, using a previously described biomolecular anchor molecule (BAM) for membranes (39, 40). Receptor–ligand interactions were successfully measured on four different cellular model systems with good reproducibility, and kinetic parameters quantifying the interactions could be estimated, opening up time-resolved interaction measurements to living suspension cells.

## Materials and Methods

### Cell Culture

All cells were cultured in a humidified incubator at 37°C and 5% CO_2_. Three laboratories participated in the present study, so as to confirm the function of the protocol in different environments and in the hands of different operators.

In Sweden, K562, Jurkat, and SKOV3 cells were maintained in RPMI 1640 cell culture medium (Biochrom AG) supplemented with 10% FBS (Sigma Life Science), 1% l-glutamine and 1% penicillin–streptomycin (=PeSt) (both Biochrom AG). For Daudi cells in addition 1% sodium pyruvate (Sigma-Aldrich) was added. A431 cells were maintained in Ham’s F10 cell culture medium (Biochrom AG) supplemented with 10% FBS (Sigma Life Science) 1% l-glutamine and 1% PeSt (both Biochrom AG).

In the USA, Ramos (ATCC^®^ CRL-1596™) cells were cultured in RPMI 1640 (ThermoFisher) supplemented with 10% FBS (Hyclone) and 1% PeSt (Gibco). HEK293 cells (used as negative control cells for experiments involving Ramos cells) were maintained in DMEM/F-12, HEPES (ThermoFisher) containing 10% FBS (ThermoFisher), 1% PeSt, 100 μg/ml Zeocin (Gibco), and 400 μg/ml Geneticin (Gibco).

In the UK, HEK293 cells stably transfected with full length FcγRIIa (131 H 27Q allotype), and the parental HEK293 cells were cultured in DMEM (Sigma Life Science) containing 10% FBS (Sigma Life Science). For real-time interaction measurements 1% PeSt (Sigma Life Science) was added.

### Cell Attachment

**Figure 1:**
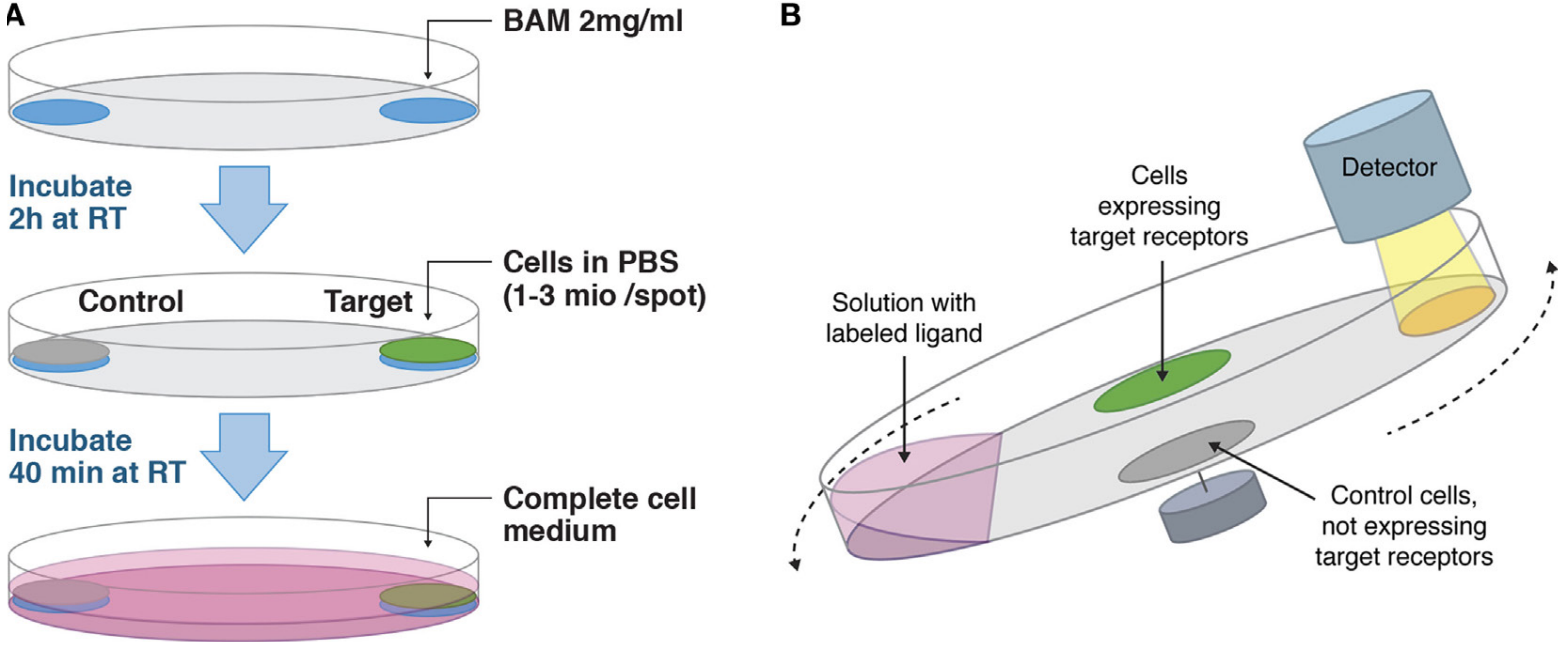
**(A)** Workflow on how to adhere cells suitable for LigandTracer assays. Target and control cells are adhered in defined areas opposite each other on a Petri dish with the help of biomolecular anchor molecule (BAM). **(B)** Measurement principle of LigandTracer. The dish containing target and control cells is placed on an inclined and rotating support. A detector is mounted above the upper part of the dish. Cell medium with labeled ligand is added to the dish and due to the inclination of the dish remains in its lower part during the measurement. The fluorescence intensity from both target and control cells is measured once per rotation in the upper position.

Biomolecular anchor molecule (SUNBRIGHT^®^ OE-040CS, NOF Corporation) was aliquoted as powder to avoid repeated freeze–thaw cycles and stored at −20°C in siliconized vials to prevent adsorption to the plastic. Directly before usage, BAM was dissolved in MQ to a concentration of 2 mg/ml. Two defined areas of a Petri dish (Nunc, Cat No 263991) were carefully covered with 400 μl of BAM solution by slowly pipetting the liquid onto the plate, so that a circular drop formed that was held in place by surface tension. The areas were about 1.5 cm in diameter and 5 mm from the rim of the dish on opposite positions (see Figure 1A). The prepared Petri dish was incubated under sterile conditions at room temperature for 2 h. For cell seeding, cells were detached with trypsin when necessary, centrifuged at 700 rpm for 5 min, and resuspended in PBS to a concentration of 2.5 or 7.5 × 10^6^ cells/ml, depending on cell size. In detail, Daudi and Jurkat cells were resuspended to 7.5 × 10^6^ cells/ml, K562, SKOV3, A431, Ramos, and HEK293 cells to 2.5 × 10^6^ cells/ml. We found that use of mechanical force while trypsinizing adherently growing cells had a negative impact on their subsequent attachment to the BAM coated dishes. Before adding the cell suspension, the remaining BAM solution was carefully aspirated from the dish, ensuring that the liquid did not exceed the defined area. Immediately after, 400 μl of cell suspension was added drop by drop onto each BAM coated spot and incubated at room temperature for 40 min. The dish was then tilted to remove the remaining suspension, and 10 ml of complete cell culture medium was added before placing the dish in the incubator.

For control measurements on adherent growing cells, A431 and SKOV3 cells were suspended to 0.33 × 10^6^ cells/ml in complete medium after trypsinizing, and 3 ml cell suspension was seeded at the lower part of inclined Nunc cell culture dishes. The dishes were kept inclined in the incubator to allow cell growth in a small portion of the dish only.

All dishes were incubated overnight in a humidified incubator at 37°C and 5% CO_2_ and used the following day for experiments.

### Cell Viability

Viability of cells incubated on BAM over night was evaluated by completely removing the cell culture medium and adding 0.4% Trypan Blue (BioRad) diluted 1:1 in PBS for 2 min. Cells were washed once with PBS before images were taken with a phase contrast microscope (Nikon).

### Antibodies and Labeling

Antihuman CD32 (FcγRIIA) (BioXcell, Clone IV.3, Cat. No. BE0224), Cetuximab (Apoteket, Sweden), Trastuzumab (Apoteket, Sweden), as well as Rituximab (Apoteket, Sweden) used for experiments with Daudi cells were labeled with fluorescein isothiocyanate (FITC).

For labeling, FITC (Sigma-Aldrich) was dissolved in DMSO to 1 μg/μl. The antibody dissolved in PBS was diluted in double the volume of borate buffer pH 9, and 100 ng FITC was added per 1 μg antibody. The reaction was incubated at 37°C for 90 min, and labeled antibody was purified through an NAP-5 column (GE Healthcare). Labeled antibody was stored at −20°C until usage.

Rituximab (Invivogen, Cat. No. huCD20-Mab1) used for experiments involving Ramos cells was labeled with Alexa-fluor 488 kit (Molecular Probes) according to the manufacturer’s instructions.

APC-labeled antihuman CD3 antibody was purchased from BioLegend (Cat. No. 300312).

### Interaction Measurements with LigandTracer

The interaction between labeled antibodies and living cells expressing a target receptor was measured in real-time with LigandTracer Green (Ridgeview Instruments). In this study, seven different LigandTracer Green instruments were used in three different laboratories.

For this assay, target cells are grown in a defined area of a Petri dish, which is placed on an inclined rotating support (see Figure 1B). The detection unit is mounted above the upper part of the dish. When adding medium containing fluorescently labeled ligand, the inclination ensures that the liquid is mainly in the lower part of the dish outside the detection area. During each full rotation, the signal from the target and a reference area is recorded. In this study, the reference area contained a negative cell line, i.e., cells that do not express the target receptor. The reference signal is automatically subtracted from the target signal, resulting in a real-time binding curve that represents specific binding of the labeled antibodies to target cells. For accurate kinetic interaction analysis, the number of receptors should remain stable throughout the experiment meaning that cells have to stay firmly attached.

Prior to kinetic measurements, the cell medium was replaced with 3 ml fresh medium. The dish was placed in LigandTracer Green equipped with either a Blue/Green or a Red/NIR detector depending on the fluorescent label used in the respective assay. All measurements were performed with 30 s detection time and 5 s detection delay; this meant that the signal from each spot was measured for 30 s and that the signal was measured 5 s after the spot was placed in the detection area to allow the medium to drain from the cells. As each dish contains a target and a reference area opposite each other, a full rotation takes a little more than 70 s resulting in a data collection frequency of 0.9 min^−1^. A baseline signal was collected for 30 min, and then labeled ligand was added in two increasing concentrations. Each concentration was incubated until sufficient curvature was obtained for subsequent extraction of kinetic parameters. Dissociation of the ligand was recorded after replacing the incubation solution with 3 ml fresh medium. Obtaining clear curvature during association measurements is crucial for robust data evaluation and is dependent on the rate constants of the interaction, the concentration and incubation time. The concentrations of the antibodies in this study were chosen by the respective laboratory where the analysis was performed, so that visible curvature was obtained within a few hours of incubation time.

### Data Analysis

Binding traces were analyzed with the evaluation software TraceDrawer 1.7 (Ridgeview Instruments). Signal levels were normalized to 0% at baseline level and 100% at the end of the second ligand incubation, respectively, to enable easy visual comparison of interaction traces. This is important as kinetic parameters are derived from the curve shape irrespectively of signal height. The normalized interaction curves were fitted to three kinetic interaction models to obtain a census fit for all replicates. The use of a “one-to-one” model is the simplest approach and describes the binding of one type of ligand to one type of target.

This “one-to-one” or Langmuir binding model assumes that two types of molecules, such as a receptor (R) and a ligand (L), interact with each other in an identical and reversible manner. This means that the binding is fully characterized by the rate of complex formation, *k*_a_, the complex dissociation rate, *k*_d_, which depends on the stability of the complex, and the concentrations of the bound and free proteins:

(1) $$[\text{R}]+[\text{L}]\overset{k_{\text{a}}}{\underset{k_{\text{d}}}{⇄}}[\text{RL}].$$

The equilibrium dissociation constant or affinity (*K*_D_) of the interaction is defined as the ratio between dissociation and association rate constants, *k*_d_/*k*_a_, and reflects the ligand concentration at which half of the receptors are occupied at equilibrium. In real-time interaction analysis, the rate constants are derived by monitoring a signal (*B*) that is proportional to the concentration of complexes [RL], while having a fixed number of receptors (*B*_max_). When incubating with a ligand concentration [L], the rate of complex formation according to Eq. 1 can be expressed as

(2) δBδt=ka⋅[L]⋅(Bmax−B)−kd⋅B,

and when the signal versus time curve contains enough curvature, the interaction rate constants (*k*_a_ and *k*_d_) and *B*_max_ can be derived.

The “one-to-two” model assumes that there are two types of target populations that the ligand binds to with different affinities. This model assumes that there are two simultaneous, independent “one-to-one” interactions. The “one-to-one two state” model represents a scenario where the ligand binds to one type of target and where the formed ligand-target complex undergoes a change to a different state from which the ligand cannot dissociate. This model assumes that the ligand-target complex can shift between the two states with distinct rate constants. A similar case to a “one-to-one-two state” model is described by the “bivalent” model, in which it is assumed that the ligand has two possible binding sites and can bind to either one or both targets.

All antibody–receptor interactions were analyzed using the “one-to-one” model. Furthermore, the interaction between FITC-Rituximab and Daudi cells was fitted to the more complex kinetic models described above. To get an estimate of how reproducible the interaction measurements were, all replicates were fitted individually to extract the main contributing on- (*k*_a_) and off- (*k*_d_) rate and then average rate constants and affinities (=*k*_d_/*k*_a_) as well as standard deviations (SD) were calculated. If interaction curves have identical incubation times, the result from simultaneously fitting an interaction model to all presented curves according to a so-called global fit is presented in all figures.

## Results

Reported values are presented as value (±SD) where applicable.

### Cell Viability and Adherence

**Figure 2:**
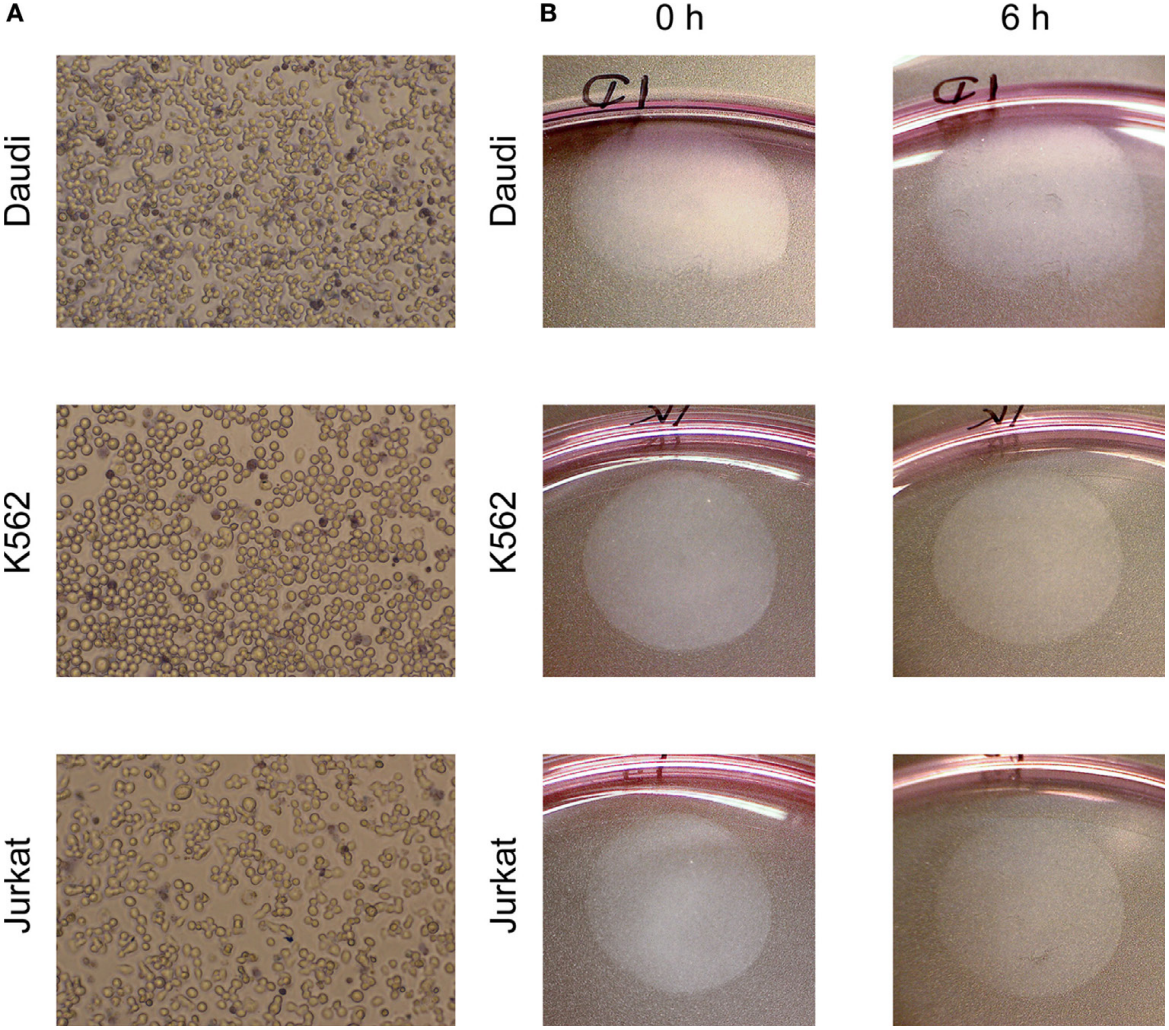
**(A)** Viability staining (Trypan Blue) of cells incubated on biomolecular anchor molecule overnight in an incubator. Dead cells are dark blue. **(B)** Cell spots, visible as round surface near the edge of a Petri dish with 10 cm diameter, before and after a 6 h LigandTracer assay with complete medium.

To verify that immune cells were viable on BAM after overnight incubation cells were stained with Trypan Blue. Representative images are shown in Figure 2A and illustrate that the majority of cells were viable. Adherence of cells was investigated by counting detached cells in the medium and imaging cell spots before and after a 6 h LigandTracer assay without ligand (Figure 2B). For Daudi and Jurkat cells, 0.6 (±0.3)% and 0.8 (±0.5)% of seeded cells were detached, respectively, whereas 11.3 (±4.3)% of K562 cells were found in the medium after a 6-h assay. Visual inspection of the cell layers confirmed that almost all cells stayed immobilized on the intended position on the cell dish.

### Analysis of the Rituximab–CD20 Interaction on Living B-Cells

**Figure 3:**
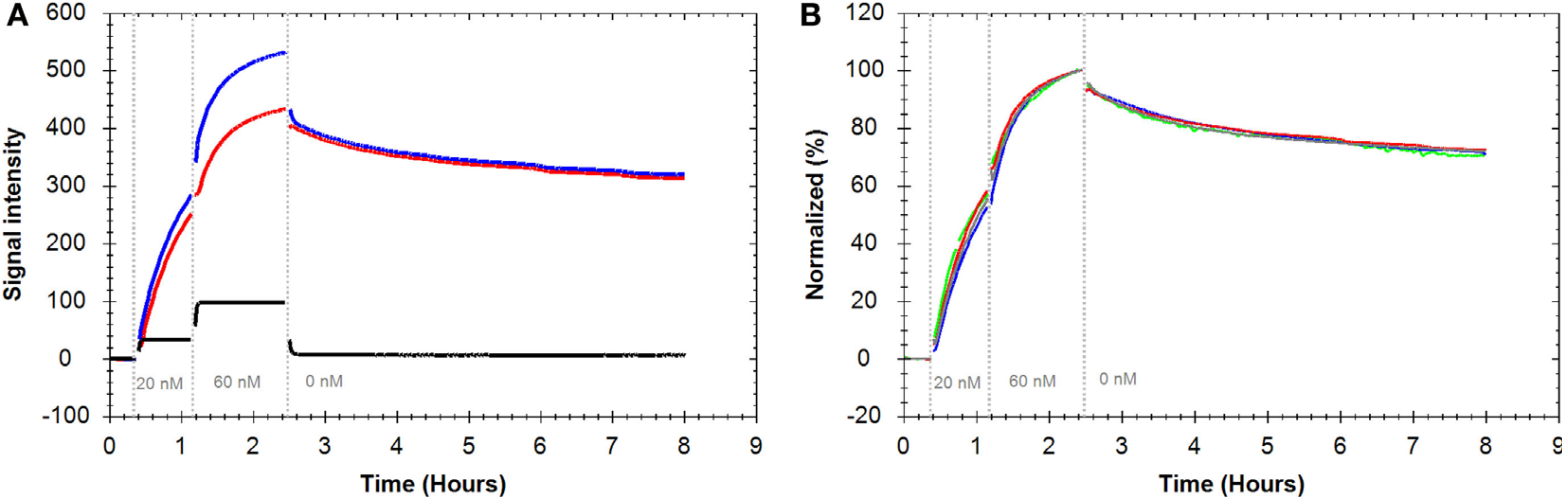
**(A)** Binding traces for fluorescein isothiocyanate (FITC)-Rituximab binding to Daudi cells (blue), K562 cells as CD20 negative control cells (black), and the subtracted trace: Daudi-K562 (red). **(B)** Binding traces for FITC-Rituximab binding to Daudi cells (*n* = 4, all reference subtracted with K562 as control cell line). Fluorescent signals were normalized to 0% at baseline level and 100% at the end of the second ligand incubation to facilitate visual comparison.

For analyzing the interaction between Rituximab and CD20, Daudi cells expressing high levels of CD20 were used as target cells whereas CD20 negative K562 cells served as control. After baseline acquisition, FITC-labeled Rituximab was added in two consecutive steps to final concentrations of 20 and 60 nM, followed by dissociation measurement in fresh medium. Introducing fluorescence to the system resulted in an immediate initial signal increase for both target and control cells (Figure 3A). This was due to unbound fluorescent ligand in the thin liquid layer present on cells during signal detection. The signal for K562 cells rapidly stabilized at a low and constant level whereas a continuous signal increase was visible for Daudi cells resulting in a curve-shaped binding trace. This illustrates that FITC-Rituximab bound specifically to B-cells.

Repeated assays produced highly similar binding traces within an 8 h time frame (Figure 3B). The reference-subtracted interaction curves were fitted to different kinetic models to extract association rate constants (*k*_a_), dissociation rate constants (*k*_d_), and the affinities, also called equilibrium dissociation constant (*K*_D_) (Table 1). Fitting a “one-to-one” model to the interaction curves resulted in an apparent affinity of 0.9 nM (±6.6%) with an on-rate (*k*_a_) of 1.4 × 10^4^ M^−1^ s^−1^ (±7.6%) and an off-rate (*k*_d_) of 1.3 × 10^−5^ (±5.2%).

**Table 1:** **Overview of the kinetic parameters estimated for the indicated interactions by applying the indicated fitting models**.

Interaction	Fitting model	*k*_a_ (M^−1^ s^−1^ × 10^4^)	*k*_d_ (s^−1^ × 10^−5)^	*K*_D_ (affinity) (nM)
Rituximab–Daudi	1:1	1.4 (±7.6%)	1.3 (±5.2%)	0.9 (±6.6%)
Rituximab–Ramos	1:1	3.5 (±2.3%)	2.4 (±3.6%)	0.7 (±1.3%)
Rituximab–Daudi	1:2	1.6 (±9.9%)	0.3 (±22.3%)	0.2 (±18.6%)
Rituximab–Daudi	1:1, 2 state	1.6 (±10.0%)	28.6 (±10.2%)	1.0 (±3.2%)
Rituximab–Daudi	Bivalent	0.9 (±5.9%)	7.3 (±6.7%)	7.8 (±5.9%)
Anti-CD3 mAb–Jurkat	1:1	8.7 (±15.0%)	4.4 (±13.4%)	0.5 (±5.4%)
Anti-CD32 mAb–HEK293_FcγRIIa	1:1	35.1 (±28.0%)	1.8 (±13.3%)	0.05 (±40.8%)

*All values are presented as value (±SD)*.

**Figure 4:**
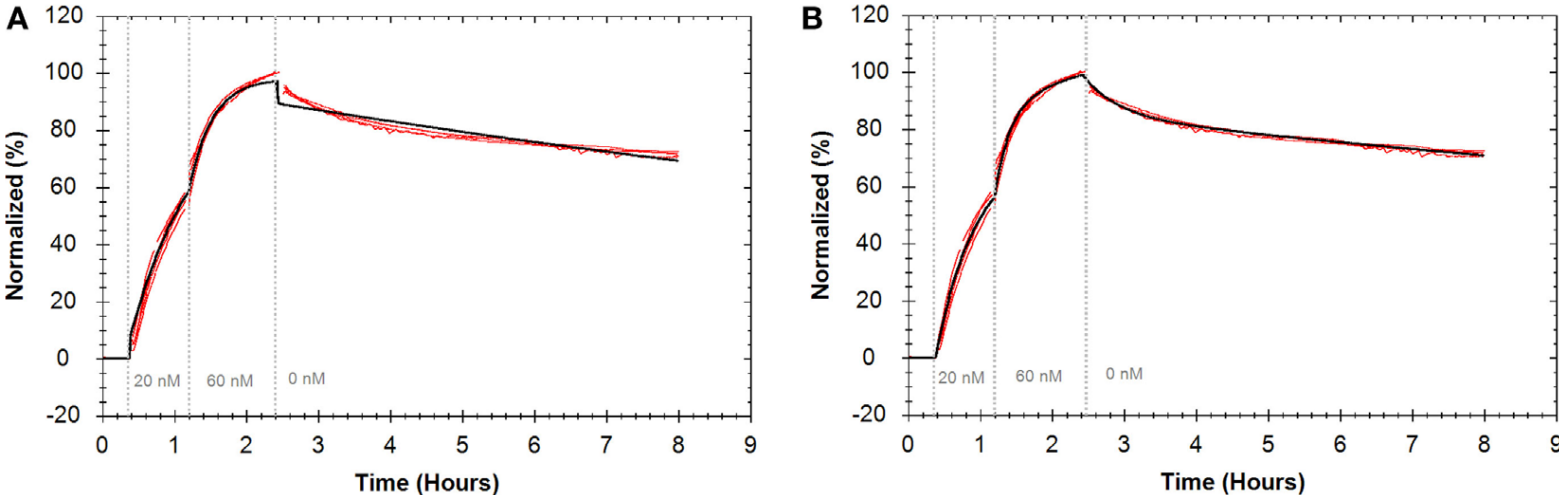
**Binding traces for fluorescein isothiocyanate-Rituximab binding to Daudi cells (*n* = 4) (red) and curves resulting from globally fitting kinetic models (black)**. For all binding traces fluorescent signals were normalized to 0% at baseline level and 100% at the end of the second ligand incubation to facilitate visual comparison. **(A)** Fit from “one-to-one” model. **(B)** Fit from “one-to-one two state” model.

The “one-to-one” model assumes that one ligand binds to one target and is the simplest modeling approach for kinetic data. As can be seen in Figure 4A, the single dissociation rate, corresponding to a single exponential signal decay, fitted to the data does not correspond well to the experimentally obtained dissociation pattern. Therefore, more complex models, such as the “one-to-two” and “one-to-one two state” model, which assume that a second process contributes to the interaction, were fitted to the data. Applying the “one-to-two” model to the Rituximab–Daudi interaction curves resulted in an apparent affinity of 0.2 nM (±18.6%) for the dominating process. Fitting a “one-to-one two state” model to those binding curves gave an apparent affinity of 1.0 nM (±3.6%). The resulting fit (black line) of the latter model is depicted in Figure 4B to exemplify that the more complex models correspond better to the measured data. The notion that Rituximab dissociation was not well described by a “one-to-one” model becomes apparent when looking at the initial 2 h of the dissociation phase (corresponding to 2.5–4 h in Figure 4B) and the remainder of the dissociation phase (corresponding to 4.5–8 h in Figure 4B). Fitting a single exponential decay to the first 2 h of the dissociation phase resulted in an off-rate of 2.3 × 10^−5^ s^−1^ (±17.0%), whereas a significantly slower off-rate of 7.4 × 10^−6^ s^−1^ (±14.3%) was obtained for the remaining dissociation phase.

Since an antibody is a binder of bivalent nature, it has two potential binding sites that can recognize the same target epitope, which in turn may lead to a higher apparent affinity (41). Applying a “bivalent” model to the Rituximab–Daudi interaction data resulted in an apparent affinity of 7.8 nM (±5.9%) for the initial binding step of Rituximab to CD20 molecule. This is approximately 10-fold lower binding strength than obtained for the same interaction analyzed according to the “one-to-one” model (Table 1), but analysis of the initial step alone leaves the avidity effect unquantified.

The interaction curves were more accurately described by the more complex models as reflected by a threefold lower chi-square value of the fit, suggesting that the process by which Rituximab bound to CD20 is more complex than a simple “one-to-one” mechanism. During data evaluation, it was noted that the “one-to-one two state” model provided the most robust fitting results, although further systematic verification is needed to unravel the exact binding mechanism.

**Figure 5:**
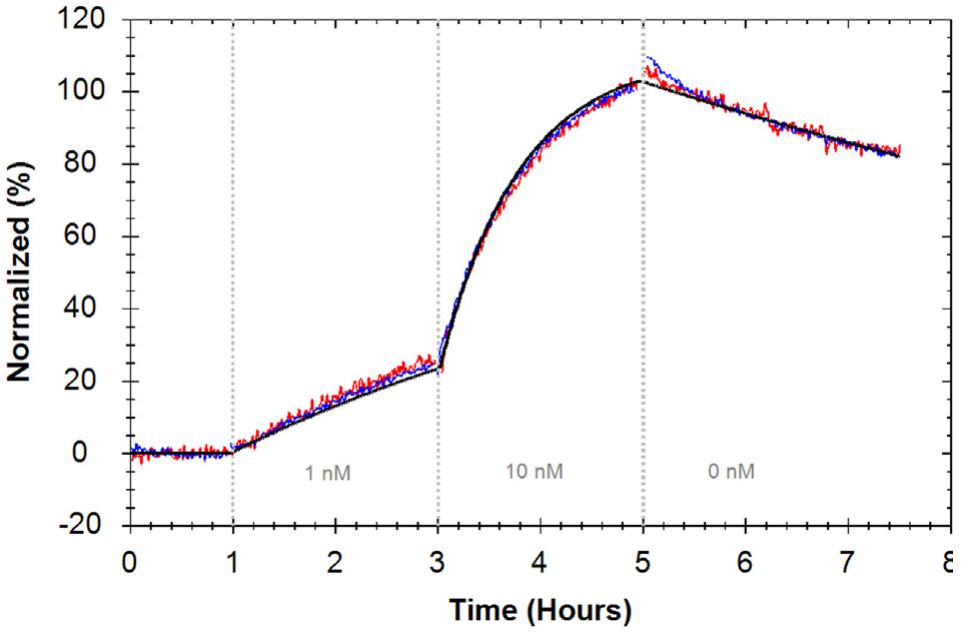
**Binding traces of Alexa488–Rituximab binding to Ramos cells (red and blue) (*n* = 2, all reference subtracted with HEK293 as control cells) and the calculated global fit according to a “one-to-one” model (black)**. Fluorescent signals were normalized to 0% at baseline level and 100% at the end of the second ligand incubation to facilitate visual comparison between binding traces.

In a different laboratory, the interaction between Rituximab and CD20 expressed on Ramos cells was analyzed with two consecutive concentrations of 1 and 10 nM Alexa488–Rituximab (Figure 5) (Table 1). The obtained apparent affinity from a “one-to-one” fit was 0.7 nM (±1.3%), which is similar to the apparent affinity observed for Rituximab binding to CD20 expressed on Daudi cells. The rate constants, however, are slightly faster for the interaction on Ramos cells (Table 1).

### BAM Does Not Influence Antibody–Receptor Interactions

**Figure 6:**
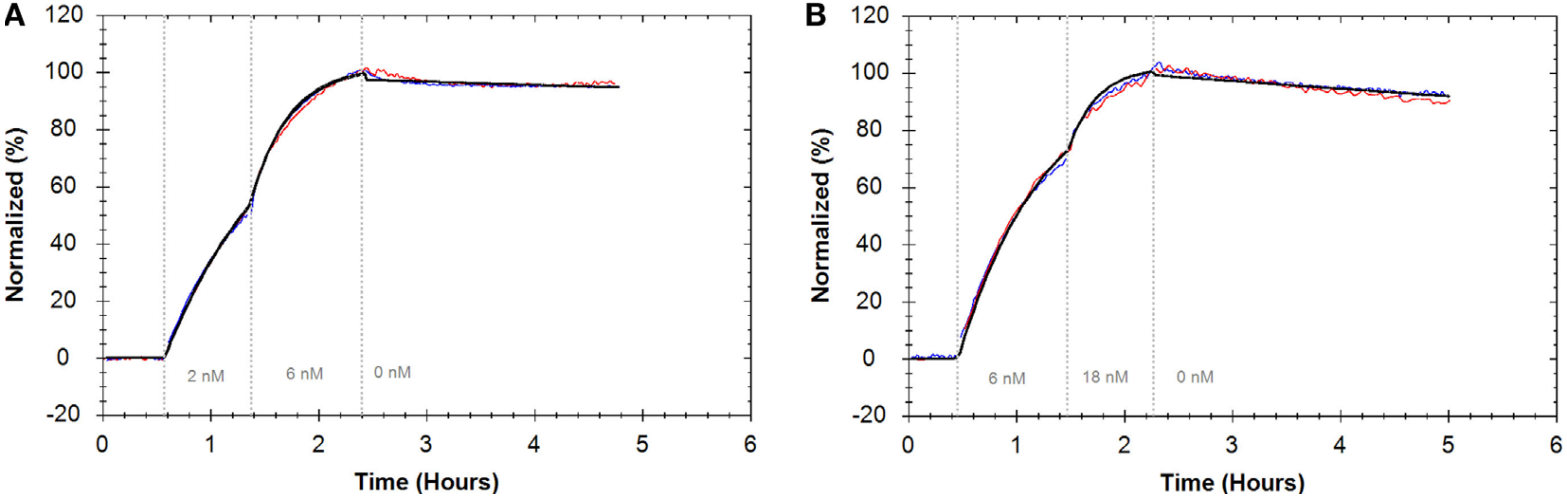
**(A)** Binding traces for fluorescein isothiocyanate (FITC)-Cetuximab binding to A431 cells intrinsically adhered on cell culture dishes (blue) and grown on biomolecular anchor molecule (BAM) (red) and the result from a global fit according to the “one-to-one” model (black). **(B)** Binding traces for FITC-Trastuzumab binding to SKOV3 cells intrinsically adhered on cell culture dishes (blue) and grown on BAM (red) and the result from a global fit according to the “one-to-one” model (black). For all binding traces, fluorescent signals were normalized to 0% at baseline level and 100% at the end of the second ligand incubation to facilitate visual comparison.

To assess if adhering cells with BAM interferes with antibody–receptor interactions on living cells, interactions were measured using two adherent growing cell lines. Binding of FITC-Cetuximab and the binding of FITC-Trastuzumab to cells seeded on tissue culture plates was compared to cells adhered with BAM. Evaluating the interactions with a “one-to-one” kinetic model resulted in an affinity of 0.02 nM for FITC-Cetuximab binding to EGFR on A431 cells in both cases (Figure 6A) (Table 2). FITC-Trastuzumab bound to HER2 with an affinity of 0.21 nM on SKOV3 cells seeded in tissue culture plates and 0.18 nM on SKOV3 cells attached with BAM (Figure 6B) (Table 2). These results imply that tethering cells with BAM generated no significant differences for interaction analysis compared to the regular approach of seeding cells on tissue culture plates. For both antibody–cell interactions, the “one-to-one” binding model reflected the data well, therefore no other models were applied.

**Table 2:** **Comparison of the kinetic parameters for interactions measured on intrinsically adhering cells versus cells tethered *via* biomolecular anchor molecule (BAM)**.

Interaction	*k*_a_ (M^−1^ s^−1^)	*k*_d_ (s^−1^)	*K*_D_ (affinity) (nM)
Cetuximab–A431 adhered	1.3 × 10^5^	2.7 × 10^−6^	0.02
Cetuximab–A431 BAM	1.4 × 10^5^	2.4 × 10^−6^	0.02
Trastuzumab–SKOV3 adhered	5.7 × 10^4^	1.2 × 10^−5^	0.21
Trastuzumab–SKOV3 BAM	6.2 × 10^4^	1.1 × 10^−5^	0.18

*All parameters were calculated by applying a “one-to-one” kinetic model*.

### Examples of Further Ligand–Immune Receptor Interactions

**Figure 7:**
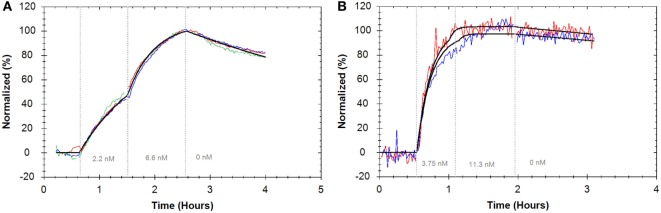
**(A)** Binding traces for APC anti-CD3 monoclonal antibody (mAb) binding to Jurkat cells (red, blue, and green) (*n* = 3, reference subtracted with K562 cells) and the result from globally fitting all curves to a “one-to-one” model (black). **(B)** Binding traces for fluorescein isothiocyanate anti-CD32 mAb binding to HEK293 cells transfected with FcγIIa receptor (red and blue) (*n* = 2, reference subtracted with non-transfected HEK293 cells). As addition of the second concentration varies slightly in time, the results from fitting a “one-to-one” model to the individual curves are displayed (black). For all binding traces, fluorescent signals were normalized to 0% at baseline level and 100% at the end of the second ligand incubation to facilitate visual comparison.

Further interactions between antibodies and different immune receptors were evaluated to demonstrate general applicability of the assay setup. In order to analyze how a commercially available APC-labeled anti-CD3 antibody bound to the CD3 T-cell co-receptor, Jurkat cells were used as target and K562 cells as control. A “one-to-one” model was fitted to the resulting binding traces (Figure 7A), resulting in an average affinity of 0.5 nM (±5.4%) (Table 1).

HEK cells are commonly used as transfection hosts, but generally they do not adhere stably enough on regular cell culture dishes for a LigandTracer assay. Therefore, as a third example, HEK293 cells stably transfected with full length FcγRIIa, were used as target cells together with the parental cell line serving as a control. The binding of a commercially available anti-CD32 antibody was fitted with a “one-to-one” model resulting in an affinity value of 0.04 nM (±40.8%) (Figure 7B; Table 1).

## Discussion

In this study, we describe and validate a method for enabling kinetic real-time measurements on living suspension cells in the time span of hours. We successfully followed ligand binding to four different suspension or weakly adhering cell lines in three different laboratories, demonstrating the robustness and general applicability of the method. The interaction between Rituximab and B-cells was analyzed in two different laboratories with different B-cell lines and different antibody concentrations and resulted in similar observed affinities of 0.7 and 0.9 nM. This further demonstrates the reproducibility of the presented protocol and the feasibility to transfer it to other laboratories. Immobilization of cells was achieved with the help of a BAM that has been described previously (39, 40). To ensure that BAM did not interfere with kinetic measurements, we used adherently growing cell lines and verified that binding of mAbs did not change when cells were grown on BAM. In total, eight cell lines were stably adhered on Petri dishes using the presented protocol.

We observed that the tested cell lines stayed well attached during the measurements, which is a prerequisite for reliable measurements in Ligand Tracer. This is particularly true for slow dissociating ligands as cell detachment interferes with accurate measurement of the off-rate. We have shown, using Rituximab as an example, that we were able to measure binding and retention reproducibly for at least 8 h. Considering that many modern therapeutics are designed to have extremely slow off-rates, it is important to provide a relevant cell-based system that enables kinetic characterization of stable binders.

The immobilized cells tolerated the assay conditions well and stayed alive during measurements. However, we noted that more liquid accumulated over the cell area during signal detection than on a cell-free reference area of the dish. When a fluorescently labeled ligand is added to the dish, a bulk effect becomes noticeable as an immediate upward jump in signal that stabilizes after a few minutes on a signal plateau. The reverse, i.e., a downward signal jump, is seen when the labeled ligand is removed during the dissociation phase. This behavior is different compared to adherent cell lines where a cell-free surface is generally suitable as reference area. To avoid confusion with fast binding processes, a negative control cell in the reference area is recommended for this suspension cell attachment protocol, especially when measuring fast interactions.

Measurements using living cells are desirable as epitopes can be changed upon chemical fixation, sometimes leading to decreased recognition by the binder. Fixation can result in underestimation of the ligand’s binding strength or even complete loss of binding to the target epitope. For example, Petrie et al. (42) described problems with a commercially available anti-CD20 antibody after PFA fixation of cells. Fixation of cells also immobilizes the receptors in the cell membrane and thus prevents processes such as oligomerization and clustering of receptors. All these processes can contribute to the biological function of a receptor and can influence the kinetics of a ligand–receptor interaction. Therefore, measuring interaction process on living cells is important to decipher the underlying mechanism of a receptor–ligand interaction (32).

LigandTracer is designed to quantify molecular interactions on living cells and by tethering suspension cells to a Petri dish this method is extended for immunological applications. Compared to biophysical techniques for real-time interaction analysis, this method offers the advantage that ligand binding to living cells can be quantified rather than measuring cellular responses (26, 27, 43). Moreover, this method allows monitoring dissociation processes slower than 1 × 10^−5^ s^−1^, which is considered the practical limit for SPR-based systems (44). As FACS can monitor multiple bound ligands to cells in suspension, it is ideally suited for multiplexed expression analysis. By sampling at various ligand incubation times, kinetic information can be extracted (45, 46). Repetitive sampling can be cumbersome, but as long as the interaction follows a “one-to-one” model, relatively few data points and only a partial dissociation phase are sufficient to extract the rate constants. When interaction processes are more complex, as observed for Rituximab binding to CD20, both the sampling rate and a full characterization of the dissociation phase as provided with the current method are beneficial.

We obtain an apparent affinity of about 1 nM for the Rituximab–CD20 interaction, which is close to values obtained by traditional saturation assays on living B-cells with Rituximab (4.4–5.5 nM) (47–49) and to values cited by the FDA (5.2–11 nM) (50). The affinities derived from cell-based assays are much higher than those obtained by biophysical systems like SPR on isolated proteins (160 nM) (51) or QCM on fixated cells (650 nM) (52). The affinity of 0.18 nM obtained for Trastuzumab is similar to reported values independent of the technology [0.23 nM for ELISA (53), 0.13 nM for SPR (53)] or the fixation of cells [0.5–5.5 nM by SPR (28) and QCM (30), depending on cell line]. For Cetuximab, the affinity obtained by LigandTracer of 0.02 nM was very close to reported values on living cells (54, 55). Estimated affinity values from studies with fixated cells [0.53 nM for QCM (29) and 0.15 nM for ELISA (56)] and isolated protein systems [0.2 nM for SPR (56)], however, are about 10-fold weaker. Nevertheless, affinity values for Cetuximab and Trastuzumab obtained on living cells do not differ as much from measurements on non-living material as they do for Rituximab. One potential explanation for the relatively large difference is that, in contrast to Cetuximab and Trastuzumab, the Rituximab–CD20 interaction does not seem to follow a simple “one-to-one” interaction model but is better described by a heterogenous binding pattern, whose underlying cause might only be present in a living system.

The heterogenous behavior is evident when looking at the dissociation, where a significantly faster off-rate was observed during the initial 2 h of the dissociation phase compared to the remaining part. If the heterogeneous Rituximab–CD20 interaction is evaluated with a “one-to-two” model, we obtain two affinity values; 0.2 nM for the stronger interaction and 16 nM for the weaker one. Since manual assays generally do not have sufficient resolution to distinguish between several contributing processes, they will report only one affinity value. In the case of Rituximab binding to living B-cells, the reported value for traditional assays lies in between the high and low affinity contribution that we measure.

There are various reasons for an interaction to become heterogeneous. One possibility is that the heterogeneity reflects the bivalent nature of an antibody. Since an antibody has two potential binding sites toward its target, the fast and slow part of the dissociation could be due to an antibody population bound with only one arm versus a population bound with both arms. Internalization is another process that can cause heterogeneity. It should be noted however, that with similar systems like Cetuximab binding to A431 cells overexpressing EGFR or Trastuzumab binding to SKOV3 cells expressing high levels of HER2, the binding curves are well described by a “one-to-one” model meaning that the measured binding process is not significantly affected by other processes. Heterogeneous binding patterns can also be due to a more complex biology of a ligand–receptor interaction. The Rituximab–CD20 interaction can, for example, be stabilized by interacting with a second target such as an Fcγ receptor or through Fc–Fc interactions. It has been suggested that slow off-rates for anti-CD20 mAbs correlate with enhanced lipid raft formation and CDC (14, 57), although this observation remains controversial and is not yet fully understood (16, 17). It is yet to be investigated whether the formation of lipid rafts stabilizes bound Rituximab or if there is another underlying cause for the complex kinetic behavior of Rituximab.

There are a plethora of different suspension cell lines, which are an essential part of the immune system, and in addition, many types of cells of various origins that are weakly adherent. With our successful results from different suspension cells and HEK cells, as conducted by different operators in different labs, we show that the reported findings are consistent and transferable. As not all immune cells are easily cultured *ex vivo*, it is of importance to use common transfection hosts, such as HEK cells when needed. The assay time of several hours was suitable to analyze modern therapeutics such as mAbs, which often display a slow dissociation rate. The cellular environment is important to study the real-life complexity of molecular interactions that involve cell-surface receptors. As one example, the interaction between Rituximab and CD20 was analyzed on living B-cells, and we could see that the binding pattern of Rituximab was heterogeneous and more complex than what is reflected by a “one-to-one” model, which has not been reported previously. In conclusion, the method presented here provides the chance to broaden the understanding of molecular interactions on a wider range of cell types, particularly within the field of immunology.

## Author Contributions

SB, KA, JBu, JR, and PR planned the work; SB, EF, JBr, PR, and JBu participated in experimental procedures and/or analyzed data; SB, KA, and JBu drafted the manuscript. All authors read, reviewed, and approved the final version of the manuscript.

## Conflict of Interest Statement

Ridgeview Instruments AB (RIAB) develops and sells the device LigandTracer, which is described in the manuscript. SB, KA, and JBu are employed by RIAB. KA and JBu are shareholders of RIAB. RIAB acknowledges the adherence to the journal policies on sharing data and materials. PR is an employee of BioRevera LLC, a company that collaborates with RIAB. JBr is an employee of Pfizer, Inc.
